# Molecular and phylogenetic study of *Staphylococcus aureus* isolated from human and cattle of Al-Qadisiyah Governorate, Iraq

**DOI:** 10.14202/vetworld.2019.1378-1382

**Published:** 2019-09

**Authors:** Ahmed Jasim Neamah, Hayder Naji Ayyez, Saba Falah Klaif, Yahia Ismail Khudhair, Muthanna Hadi Hussain

**Affiliations:** 1 Unit of Zoonotic Diseases, College of Veterinary Medicine, University of Al-Qadisiyah, Al-Qadisiyah, Iraq; 2 Department of Internal and Preventive Medicine, College of Veterinary Medicine, University of Al-Qadisiyah, Al-Qadisiyah, Iraq

**Keywords:** antibiotic, leukotoxin, methicillin, Panton-Valentine, resistance, *Staphylococcus aureus*

## Abstract

**Aim:**

This study was designed to detect the prevalence of *Staphylococcus aureus*, to estimate the frequency of methicillin resistance gene (*mecA*), *femA* (specific gene for *S. aureus*), and *lukS* gene, and the prevalence of urinary tract infection (UTI) in human and bovine mastitis caused by *S. aureus*.

**Materials and Methods:**

A total of 102 cases of *S. aureus* were included in this study; 72 specimens were isolated from human with UTIs and 30 specimens were isolated from milk of cattle with acute mastitis. Diagnosis was done by VITEK 2 Compact after subculture and purification. All isolates were examined for the presence of *mecA*, *femA*, and *lukS* (Panton-Valentine leukocidin) using multiplex polymerase chain reaction.

**Results:**

Culture and biochemical evaluation of the samples revealed the presence of *S. aureus*, among which the genes *mecA, femA*, and *lukS* were positively detected in 68 (94.4%), 36 (50%), and 20 (27.7%) of *S. aureus* isolates from methicillin-resistant humans, respectively. In the same manner, the genes *mecA, femA*, and *lukS* were positively detected in 27 (90%), 14 (46.7%), and 11 (36.7%) of *S. aureus* isolates from methicillin-resistant cattle. Sequencing of partial order of *femA* gene isolated from human isolate and from cattle with *mecA* isolated from human revealed high sequence identity with the National Center for Biotechnology Information (NCBI)-Basic Local Alignment Search Tool. *S. aureus* isolates and the phylogenetic analysis showed that there was a significant genetic similarity (0.5 genetic change) between human and animals isolates, and then, the gene sequences were deposited into NCBI-Genbank accession numbers MG696860.1 for *mecA* and *femA* from human, MG696861.1 for *mecA* and *femA* from cattle, MK474469.1 for *mecA* and *femA* gene from human, and MG696862.1 for *mecA* and *femA* gene from cattle.

**Conclusion:**

The study represents the first report of genetic relationship between *S. aureus* from humans and cattle of Iraq. Therefore, it is essential to define the role of animals as an important source of the distribution of pathogen related to public health. The continuous monitoring of methicillin susceptibility pattern of *S. aureu*s isolates that have high standards of infections might prevent methicillin-resistant *S. aureu*s transmission in either direction between human and cattle, the risk of dairy milk on humans, or self-direction between the same species.

## Introduction

Since the discovery and extensive utilization of antibiotics, multidrug-resistant strain of *Staphylococcus aureus* has emerged. Originally, methicillin-resistant *S. aureus* (MRSA) was discovered in hospitals in the 1960s, which then spread worldwide in community and hospitals in the 1990s, creating reservoirs in both settings [1]. Various infections, ranging from minor skin infections to severe diseases, such as urinary tract infections (UTIs), toxic shock, septicemia, and endocarditis, could be caused by *S. aureus*. The adaptive power of *S. aureus* to antibiotics produced to the emergence of MRSA. MRSA strain is characterized by its potential to express many virulence factors such as Panton-Valentine leukocidin (PVL). Thus, MRSA is one of the important pathogens implicated in hospital-acquired infection. *S. aureus* is an important pathogen responsible for both nosocomial and community-acquired infections in people and also causes common diseases in animals, especially mastitis. This ubiquitous organism is carried asymptomatically in 20-30% of the human population [2].

It can lead to a wide range of maladies such as skin and soft tissue infections, mastitis, bone, joint and implant infections, pneumonia, and septicemia. It might be the cause of similar diseases in animals; however, is most economically significant as a cause of bovine mastitis (BM) [3]. The extensive use of antibiotics that accounted for the hasty dissemination of antibiotic resistance in *S. aureus* has become a global concern; the most notable example of this phenomenon is MRSA. The first clinical isolate of MRSA was confirmed in 1961, just 1 year after the launch of methicillin, and in the 1980s, it became a widespread health-care concern [3,4]. Methicillin resistance may be attributed to a penicillin-binding protein (PBP) 2a with an altered β-lactam affinity [5,6]. The *mecA* gene encodes for PBP 2a protein, which is carried by a mobile element “Staphylococcal chromosomal cassette (SCC)” and used as a molecular marker for MRSA [7]. Simultaneously, certain factors essential for methicillin resistance (*fem*) [8] are found in both methicillin-susceptible and MRSA. The part of *fem’s* factor that combines physically distant from SCC affects the level of the resistance [9]. Integration of the *mec* complex and neighboring regions into an SCC element was one of the factors contributing to the evolution of *mecA*-mediated β-lactam resistance in Staphylococci [10]. MRSA was phenotypically detected in 68.7% of samples obtained from cattle with mastitis while the methicillin-susceptible *S. aureus* was detected in 31.3% of these samples. Genotypic analysis revealed that 54.5% phenotypically methicillin resistance isolates were *mecA* gene positive, while 45.5% of the isolates were *mecA* gene negative [11]. This organism is one of the important pathogens in hospital-acquired infection. It has developed resistance to a wide range of antimicrobial drugs, which complicates the treatment of infections. In particular, MRSA has become a disreputable etiological agent for several infections and among the foremost necessary health facility pathogens worldwide [12]. *S. aureus* is characterized by its several specific virulence factors. The PVL is a major virulence factor related to death lesions of the skin and body covering tissues (e.g., furuncles) and also with community-acquired severe necrotic pneumonia [13]. In humans, *S. aureus* is found in the anterior nares. Nasal carriage of these organisms in hospital staff is a potential source for infection in hospitalized patients, and elimination of nasal carriage has been reported to reduce the incidence of *S. aureus* infections. The antimicrobial resistance profile of human *S. aureus* is of great public health concern, especially in developing countries where health facilities are inadequate [14].

This study aimed to detect the prevalence of *S. aureus* and the frequency of methicillin-resistant genes and the prevalence of UTI in human and BM caused by *S. aureus*.

## Materials and Methods

### Ethical approval

The Animal Ethical Committee of the College of Veterinary Medicine, University of Al-Qadisiyah, Iraq, has approved this study.

### Samples

Samples were obtained from 72 individuals suffering from UTI at the hospital in Al-Qadisiyah Governorate during January 2016-March 2016, and at the same time, samples were also obtained from 30 cows having acute mastitis.

### Bacterial diagnosis

Bacterial isolates were identified based on cultural and biochemical criteria. The method followed was adapted from a previous study that reported that VITEK2 cards inoculated with fluids sampled directly from culture bottles are suitable for speedy identification and susceptibility testing [15]. A sterile swab or applicator stick was used to transfer a sufficient number of colonies of a pure culture in 3.0 mL of sterile saline (0.45% NaCl, pH 4.5-7.0) in a 12×75 mm clear plastic (polystyrene) test tube. The turbidity was adjusted to 0.50-0.63 Mf and measured using a turbidity meter known as DensiChek [15]. Results were read overnight.

### Molecular detection of virulence genes

Multiplex polymerase chain reaction (PCR) was performed to amplify target genes using primers for the following genes: *femA* (specific for *S. aureus*), *mecA* gene (encoding PBP 2a), and *lukS* (responsible for the production of PVL). The sequences of these primers are listed in Table-1 [16,17].

**Table 1:** Sequences of primers used for multiplex PCR.

Product genes	Primer name	5’–3’	Size (bp)	Reference
*femA*	*femA-F* *femA-R*	CGATCCATATTTACCATATCA ATCACGCTCTTCGTTTAGTT	450	[17]
*mecA*	*mecA-F* *mecA-R*	ACGAGTAGATGCTCAATATAA CTTAGTTCTTTAGCGATTGC	293	
*lukS*	*lukS-F* *lukS-R*	CAGGAGGTAATGGTTCATTT ATGTCCAGACATTTTACCTAA	151	

PCR=Polymerase chain reaction

### DNA extraction of bacterial genome

Fresh bacterial genomic DNA of *S. aureus* was extracted from nutrient broth samples using Presto™ Mini Genomic DNA Bacteria Kit (Geneaid, China), according to a previously described method [17]. The extracted DNA was validated by Nanodrop spectrophotometer and stored at −20°C in the refrigerator until further usage.

### Polymerase chain reaction (PCR)

The *femA* gene (specific to *S. aureus* species), *mecA* gene (encoding PBP 2a), and *lukS* gene (responsible for the production of PVL) were detected using multiplex PCR. The primers were prepared by Bioneer, Korea.

### DNA sequencing

DNA sequencing was performed for confirmative detection of *S. aureus* strain; based on positive detection of *femA* and *mecA*, two isolates were obtained from humans and one from cattle, and the respective PCR products were purified from agarose gel. The purified *femA* and *mecA* fractions were sent to Bioneer, Korea, for DNA sequencing using Applied Biosystems DNA sequencing system. The genomic sequences were assembled and submitted in GenBank-National Center for Biotechnology Information followed by multiple sequence alignment using Basic Local Alignment Search Tool for phylogenetic tree construction and phylogenetic analysis by MEGA10 program (https://www.megasoftware.net/).

## Results

**Figure-1:**
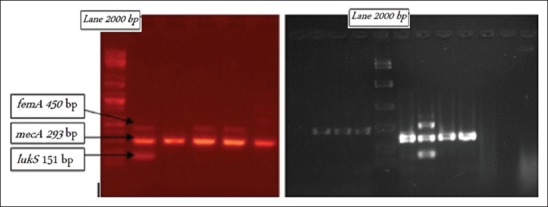
Multiplex polymerase chain reaction for the detection of three genes: *femA* (450 bp), *mecA* (293 bp), and *lukS* (151 bp).

Culture and biochemical evaluation of the samples revealed the presence of *S. aureus* with positive detection of *mecA, femA*, and *lukS*, respectively, in 68 (94.4%), 36 (50%), and 20 (27.7%) of samples isolated from methicillin-resistant human samples. Similarly, *mecA, femA*, and *lukS* were positively detected, respectively, in 27 (90%), 14 (46.7%), and 11 (36.7%) of samples isolated from methicillin-resistant cattle. The MRSA was confirmed by the detection of *mecA* gene, considered as a genotyping marker, in 68 of 72 (94.4%) samples, while the remaining four isolates were methicillin-sensitive *S. aureus*, although these were methicillin-resistant phenotypically. The multiplex PCR revealed significant differences in the band sizes of *mecA, femA*, and *lukS* (450, 293, and 151 bp, respectively) as illustrated in Figure-1.

**Figure-2:**
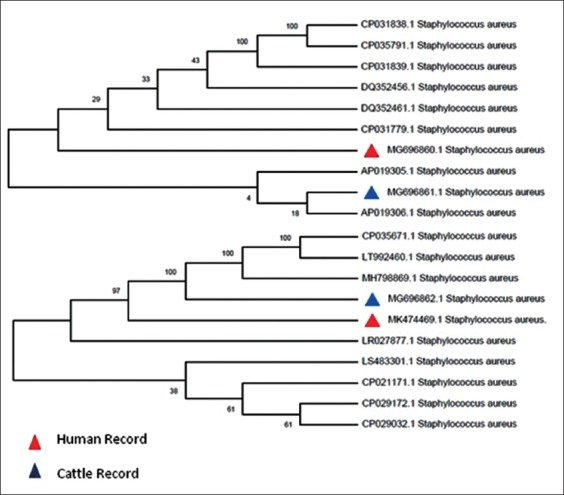
Genetic tree interpretation based on *mecA and femA* gene partial sequences used for methicillin-resistant *Staphylococcus aureus* genetic changes according to host and regions.

The results of nucleotide sequencing of human and cattle MRSA isolates revealed the sequences of targeted genes (*mecA* and *femA* partial gene) that were recorded in Genbank with accession numbers MG696860.1 and MK474469.1 for human isolates and with accession numbers MG696861.1 and MG696862.1 for bovine milk isolates, respectively. Comparative analysis of nucleotide sequences from Iraq samples with the number of global strains present in the Genbank database is shown in Figure-2.

## Discussion

*S. aureus* has been recognized as an important pathogen that causes serious problems in community and hospitalized patients [18]. UTI is the most consequence of common community and hospital bacterial infection and characterized by high rate of treatment failure and high prevalence of *S. aureus* [19].

About 36.3% of clinical samples isolated from patients with respiratory tract infections in Shiraz province were positive for MRSA, among which the most commonly detected antibiotic resistance genes were *tetK* (89.18%) and *mecA* (71.62%) [20], which suggested that MRSA has more affinity to the UIT than the respiratory infection with the similarity in the presence of the résistance genes, especially *mecA*. In a total of 152 studies on the community-associated MRSA; the highest prevalence was found in India (16.5-23.5%) followed by Vietnam (7.9%) and Taiwan (3.5-3.8%) [21]; the difference of these countries from Iraq may be attributed to the cruel circumstances of people in Iraq through the past few decades. A previous study showed that 81 MRSA strains (71.05%) were *mecA* gene positive [22], which was slightly less than the results of our study (94.4%); it may be because, for the previous study, samples were obtained from multilocal lesions; nasal swab, burns, and urine, while we obtained the samples from only UTI in our study.

On the other side, MRSA is highly prevalent in cattle causing mastitis, the most common cattle infection, which leads to a huge economic loses. Hence, the use of right antibiotic in the treatment and control of BM caused by *S. aureus* is crucial [11]. This corroborated with the results found in our study, in which *S. aureus* was detected. This study focused on mastitis associated with *S. aureus* as an important cattle disease with a high incidence in buffaloes, as reported previously [23,24].

This study was designed to provide information about the distribution of MRSA based on genotypic marker, *mecA*, among UTI patients in Al-Qadisiyah Governorate and phylogenic relationship with MRSA isolated from milk obtained from cattle. The gene *mecA* was detected in 94.4% of oxacillin-resistant *S. aureus* [18] which reveals it to be a potential genotypic marker for this criterion.

Among 72 isolates used in this study, 50% of the isolates did not harbor the gene *femA* and these isolates might possess *femB* marker. The results revealed that 29.4% of *mecA*-positive samples expressed *lukS* gene and all were positive for *femA*, indicating the correlation between the presence of *lukS* and the presence of *femA* because all the isolates that did not possess *femA* also lacked *lukS* gene. There was high variation for this gene among MRSA isolates. In India, a previous study reported that 85.1% of MRSA expressed PVL gene [25], while another study reported a prevalence of 64% for *lukS* gene among MRSA samples [26]. Such differences in the results were attributed to the geographical variation.

To determine the degree of relatedness between the two clinically isolated local strains of *S. aureus* from human and cattle, phylogenetic trees for *femA* and *mecA* were constructed based on each gene separately (Figure-2). The sequences of *femA* from the two clinically isolated animal and human strains belonged to Clade I and Clade II, respectively. On the contrary, *mecA* sequences from the two clinically isolated animal and human strains belonged to the same clade. The present data suggested the novelty of the two clinically isolated animal and human strains. This result indicates the indispensable need to employ an Multilocus sequence type (ST) approach in the future analyses to assign the ST of each strain and the likelihood of raising a novel ST from the strains under study.

## Conclusion

*S. aureus* is recognized as an important pathogen that causes serious problems in community, hospitalized patients, and animals. The present data suggested the novelty of the two clinically isolated local strains from animal and human. Consequently, the continuous monitoring of methicillin susceptibility pattern of *S. aureus* isolates that have high standards of infections might help in the prevention of MRSA transmission in either direction between human and cattle, the risk of dairy milk on humans, or self-direction between the same genus.

## Authors’ Contributions

YIK and HNA proposed the hypothesis. AJN designed the study. AJN and HNA collected the samples from human while the samples were collected from cattle by YIK and MHH. AJN and SFK conducted the experiments. All authors collected the references, read and approved the final manuscript after editing by MHH.
